# Supplementary material for: Structure and dynamics of the pyroglutamylated RF-amide peptide QRFP receptor GPR103
Source: Nat Commun. 2024 Jun 19;15:4769. doi: 10.1038/s41467-024-49030-5 (PMC11187126; doi:10.1038/s41467-024-49030-5)
Supplement: Supplementary file 1 — Supplementary Information [file 41467_2024_49030_MOESM1_ESM.pdf]

TGFα shedding response	WT (1:1)	WT (1:2)	WT (1:5)	WT (1:10)	WT (1:20)	WT (1:50)	Δ1-18	Δ1-30	Δ1-43	ΔECL2	ΔCterm	F11$^{\text{N-term}}$A
n =	5	5	5	5	5	3	3	3	3	3	3	3
pEC50	8.29 ± 0.13	8.18 ± 0.12	7.9 ± 0.15	7.82 ± 0.12	7.67 ± 0.13	7.27 ± 0.03	NA	NA	NA	NA	7.95 ± 0.02	6.75 ± 0.05
(EC$_{50}$)	(5.1 nM)	(6.6 nM)	(12 nM)	(15 nM)	(21 nM)	(54 nM)	NA	NA	NA	NA	(11 nM)	(180 nM)
Emax	100.0 ± 0	102.3 ± 1.4	99.4 ± 1.7	86.5 ± 1.5	69.6 ± 1.9	38.9 ± 2.7	NA	NA	NA	NA	89.2 ± 1.5	103.8 ± 3.0

TGFα shedding response	F11$^{\text{N-term}}$A	L14$^{\text{N-term}}$A	F25$^{\text{N-term}}$A	I26$^{\text{N-term}}$A	Y29$^{\text{N-term}}$A	C98$^{2.57}$A	T102$^{2.61}$A	Q105$^{2.64}$A	W111$^{\text{ECL1}}$A	C118$^{3.25}$A	Q125$^{3.32}$A	V129$^{3.36}$A
n =	3	3	3	3	3	3	3	3	3	3	3	3
pEC50	6.75 ± 0.05	8.12 ± 0.03	7.10 ± 0.06	7.36 ± 0.12	6.57 ± 0.06	7.14 ± 0.04	7.69 ± 0.04	8.43 ± 0.08	NA	NA	6.55 ± 0.10	6.87 ± 0.10
(EC$_{50}$)	(180 nM)	(7.5 nM)	(79 nM)	(44 nM)	(270 nM)	(72 nM)	(21 nM)	(3.7 nM)	NA	NA	(280 nM)	(130 nM)
Emax	103.8 ± 3.0	105.7 ± 2.1	109.6 ± 1.2	97.6 ± 5.0	94 ± 2.9	88.3 ± 3.6	105.0 ± 3.6	96.8 ± 1.5	NA	NA	35.7 ± 5.1	96.2 ± 2.3

TGFα shedding response	Q125$^{3.32}$A	V129$^{3.36}$A	Q184$^{4.64}$A	L193$^{\text{ECL2}}$A	Y194$^{\text{ECL2}}$A	C201$^{\text{ECL2}}$A	E203$^{\text{ECL2}}$A	W205$^{\text{ECL2}}$A	Y214$^{5.38}$A	T215$^{5.39}$A	F282$^{6.44}$A	C285$^{6.47}$A
n =	3	3	3	3	3	3	3	3	3	3	3	3
pEC50	6.55 ± 0.10	6.87 ± 0.10	6.63 ± 0.08	6.74 ± 0.07	7.03 ± 0.08	NA	6.54 ± 0.05	NA	6.98 ± 0.06	8.19 ± 0.16	NA	7.85 ± 0.13
(EC$_{50}$)	(280 nM)	(130 nM)	(240 nM)	(180 nM)	(94 nM)	NA	(290 nM)	NA	(100 nM)	(6.5 nM)	NA	(14 nM)
Emax	35.7 ± 5.1	96.2 ± 2.3	102.9 ± 2.2	93.8 ± 1.8	102.0 ± 6.1	NA	101 ± 0.94	NA	16.6 ± 2.2	103.0 ± 2.2	NA	86.7 ± 2.0

TGFα shedding response	F282$^{6.44}$A	C285$^{6.47}$A	W286$^{6.48}$A	F289$^{6.51}$A	E297$^{6.59}$A	Q318$^{7.39}$A	F322$^{7.43}$A	C327$^{7.48}$A	E203$^{\text{ECL2}}$A /E297$^{6.59}$A	E203$^{\text{ECL2}}$A /Q211$^{5.35}$E /E297$^{6.59}$A
n =	3	3	3	3	3	3	3	3	3	3
pEC50	NA	7.85 ± 0.13	5.95 ± 0.02	NA	7.55 ± 0.11	7.07 ± 0.05	7.61 ± 0.11	8.37 ± 0.18	NA	6.37 ± 0.08
(EC$_{50}$)	NA	(14 nM)	(1100 nM)	NA	(28 nM)	(85 nM)	(25 nM)	(4.3 nM)	NA	(430 nM)
Emax	NA	86.7 ± 2.0	75.7 ± 1.9	NA	111.9 ± 1.3	11.7 ± 1.3	42.7 ± 2.8	103.5 ± 2.3	NA	108.8 ± 2.9

Supplementary Table 1 | Pharmacological characterization of mutant receptors.
Source data are provided as a Source Data file.

**Data collection**	GPR103-Gq (refined)	GPR103-Gq (upright)	GPR103-Gq (tilted)
Microscope	Titan Krios (Thermo Fisher Scientific)		
Voltage (keV)		300	
Electron exposure (e^-/Å^2)		49.6	
Detector	Gatan K3 Summit camera (Gatan)		
Magnification		×105,000	
Defocus range (µm)		-0.8–1.6	
Pixel size (Å/pix)		0.83	
Number of movies		9,555	
Symmetry		C1	
Picked particles		11,085,317	
Final particles		142,831	
Map resolution (Å)		3.19	
FSC threshold		0.143	
**Model refinement**			
Atoms			
**R.m.s. deviations from ideal**			
Bond lengths (Å)	0.003	0.003	0.003
Bond angles (°)	0.591	0.558	0.615
Validation			
Clashscore	11.98	12.34	13.8
Rotamers (%)	0.1	0	0
**Ramachandran plot**			
Favored (%)	97.22	95.98	95.67
Allowed (%)	2.78	4.02	4.33
Outlier (%)	0	0	0

Supplementary Table 2 | **Cryo-EM data collection, refinement and validation statistics**.

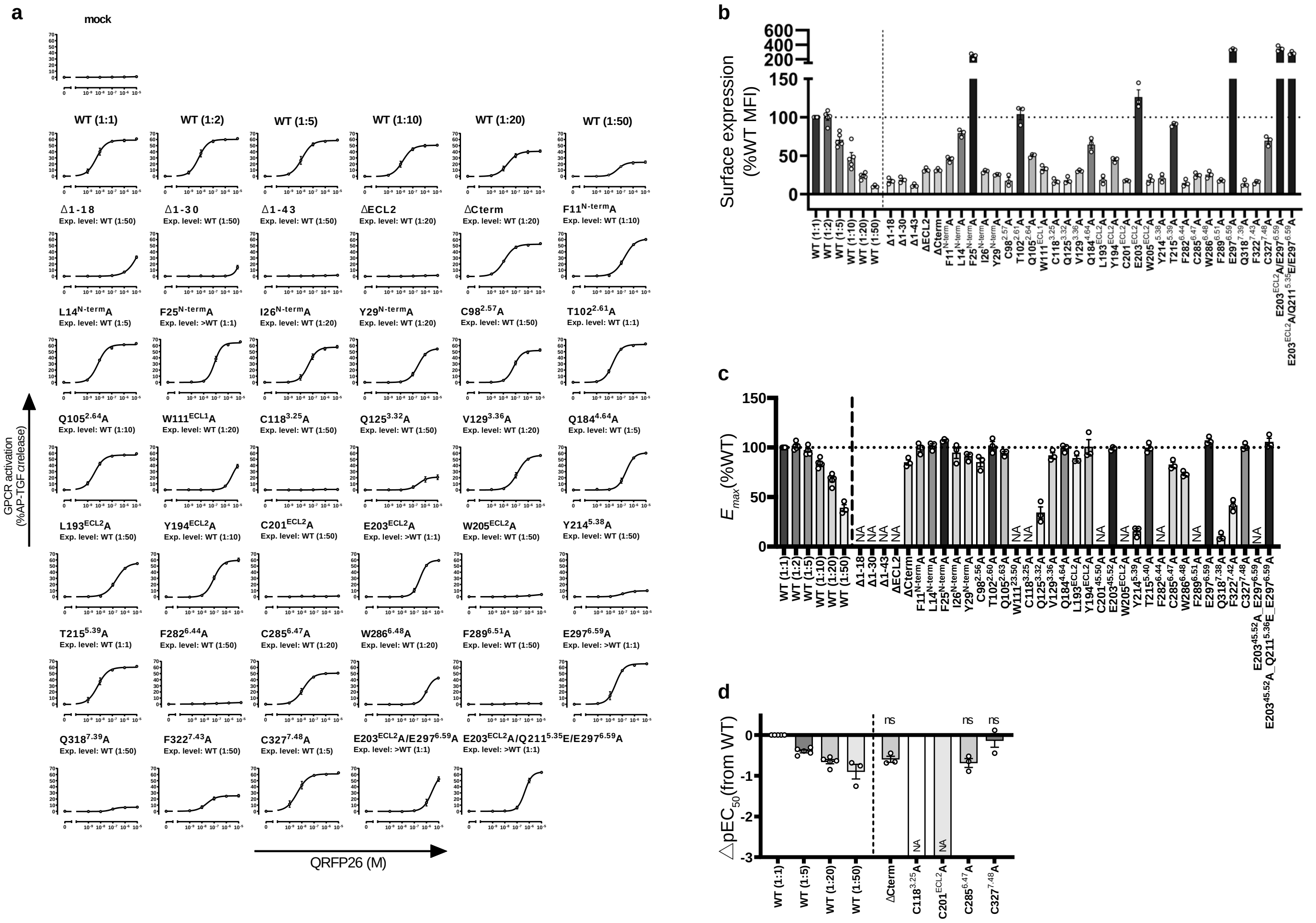

Supplementary Figure 1 | Mutational analysis of GPR103.

a Concentration-response curve of AP-TGFα release response upon QRFP26 treatment of HEK293 cells expressing the wild-type GPR103 or the GPR103 mutants. Data are presented as mean values ± SEM, normally *n*=3-5; dots), each performed in triplicate. b-d Cell-surface expression (b) and QRFP26-induced activation of GPR103 (c, d) were analyzed by the flow cytometry and the TGFα shedding assay, respectively. From the concentration–response curves (a), Emax (c) and ΔpEC50 (d) values relative to the wild type were calculated. Colors in the mutant bars indicate an expression level matching to that of titrated wild type. Statistical analyses were performed using the ordinary one-way ANOVA followed by Dunnett tests with the expression-matched (colored) WT response. ns, $p > 0.05$; *$p < 0.05$; **$p < 0.01$; ***$p < 0.001$. NA, parameter not available because of lack of the ligand response. Data are presented as mean values ± SEM (*n*=3-5; dots).Source data are provided as a Source Data file.

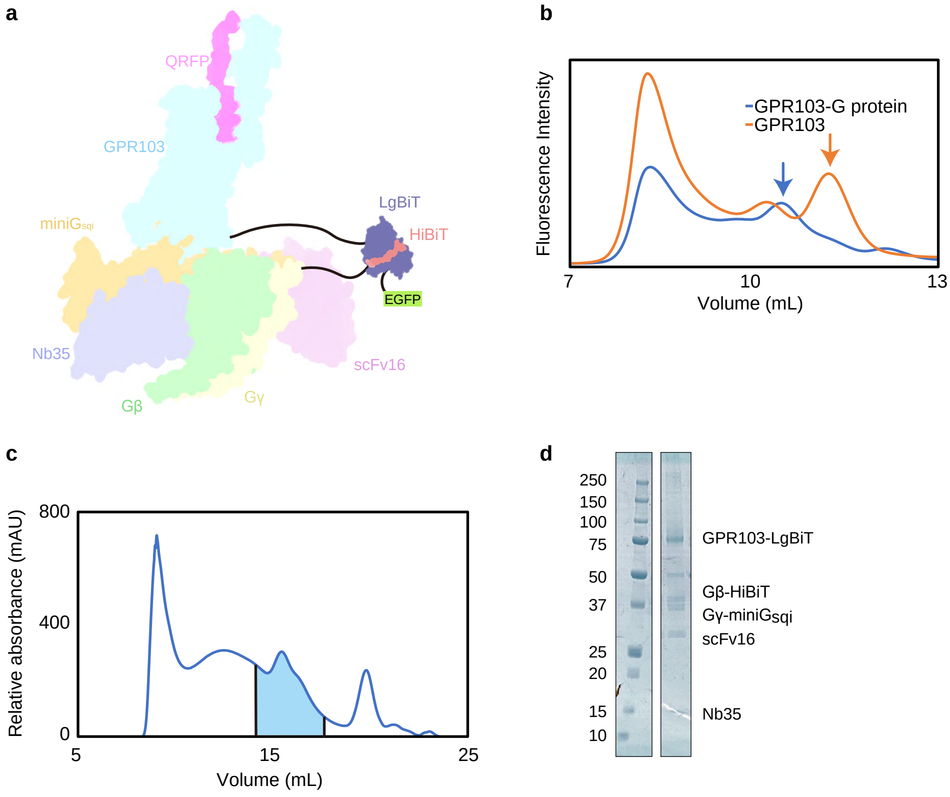

Supplementary Figure 2 | Sample purification.

a Schematic representations of the fusion-G system. **b** Fluorescence-detection size-exclusion chromatography (FSEC) analysis of complex formation by the GPR103. Solubilised cells expressing only the GPR103 are orange, and co-expressing the GPR103 and G-protein are blue.Source data are provided as a Source Data file. **c** Size-exclusion chromatography of the GPR103-G-protein complex on Superose 6 increase column. The blue fraction was collected. Source data are provided as a Source Data file. **d** SDS-PAGE gel of samples after Size-exclusion chromatography.Source data are provided as a Source Data file.

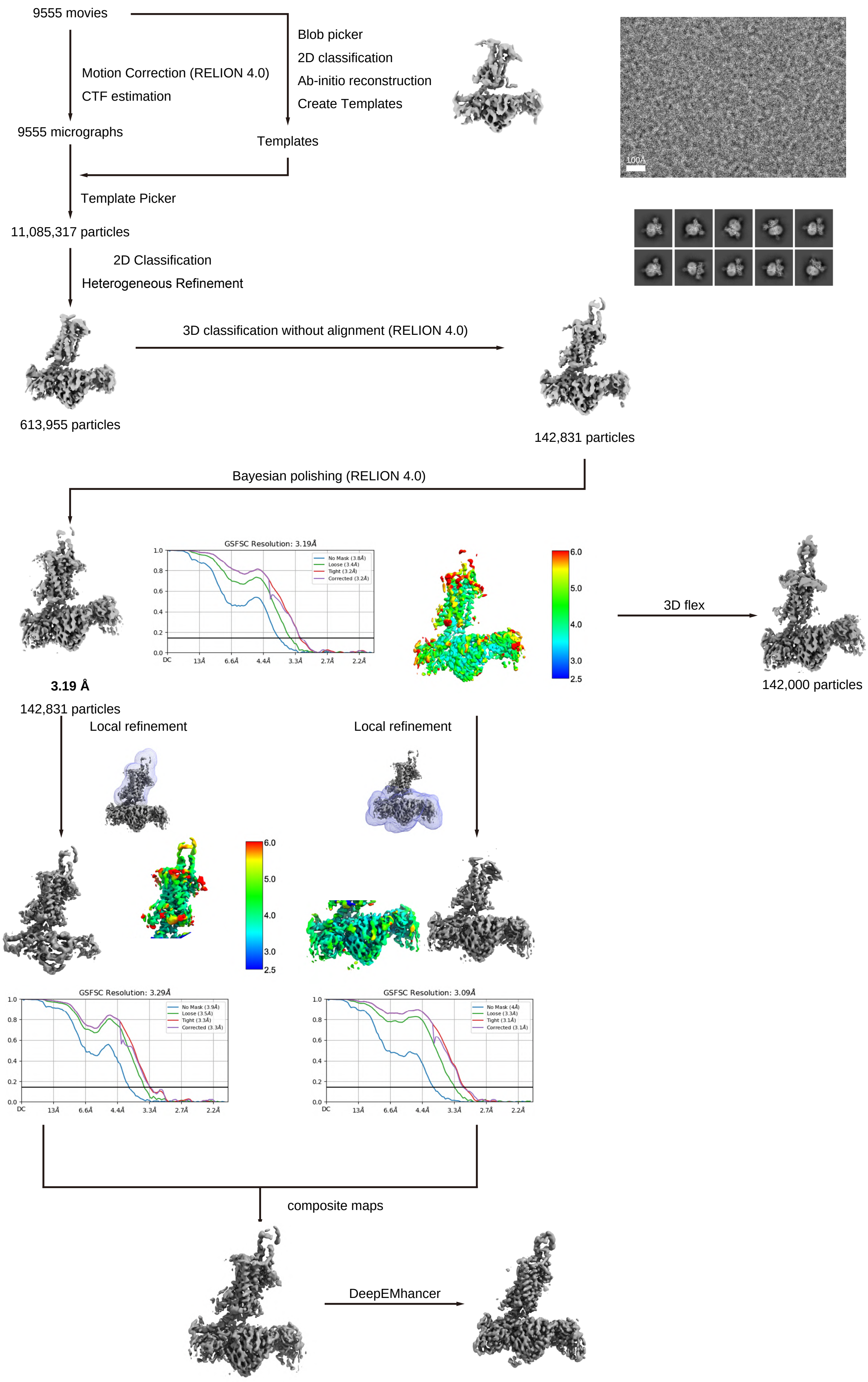
9555 movies
Motion Correction (RELION 4.0)
CTF estimation
9555 micrographs
Blob picker
2D classification
Ab-initio reconstruction
Create Templates
Templates
100Å
Template Picker
11,085,317 particles
2D Classification
Heterogeneous Refinement
613,955 particles
3D classification without alignment (RELION 4.0)
142,831 particles
Bayesian polishing (RELION 4.0)
GSFSC Resolution: 3.19Å
No Mask (3.8Å)
Loose (3.4Å)
Tight (3.2Å)
Corrected (3.2Å)
3.19 Å
142,831 particles
3D flex
142,000 particles
Local refinement
Local refinement
GSFSC Resolution: 3.29Å
No Mask (3.9Å)
Loose (3.5Å)
Tight (3.3Å)
Corrected (3.3Å)
GSFSC Resolution: 3.09Å
No Mask (4Å)
Loose (3.3Å)
Tight (3.1Å)
Corrected (3.1Å)
composite maps
DeepEMhancer

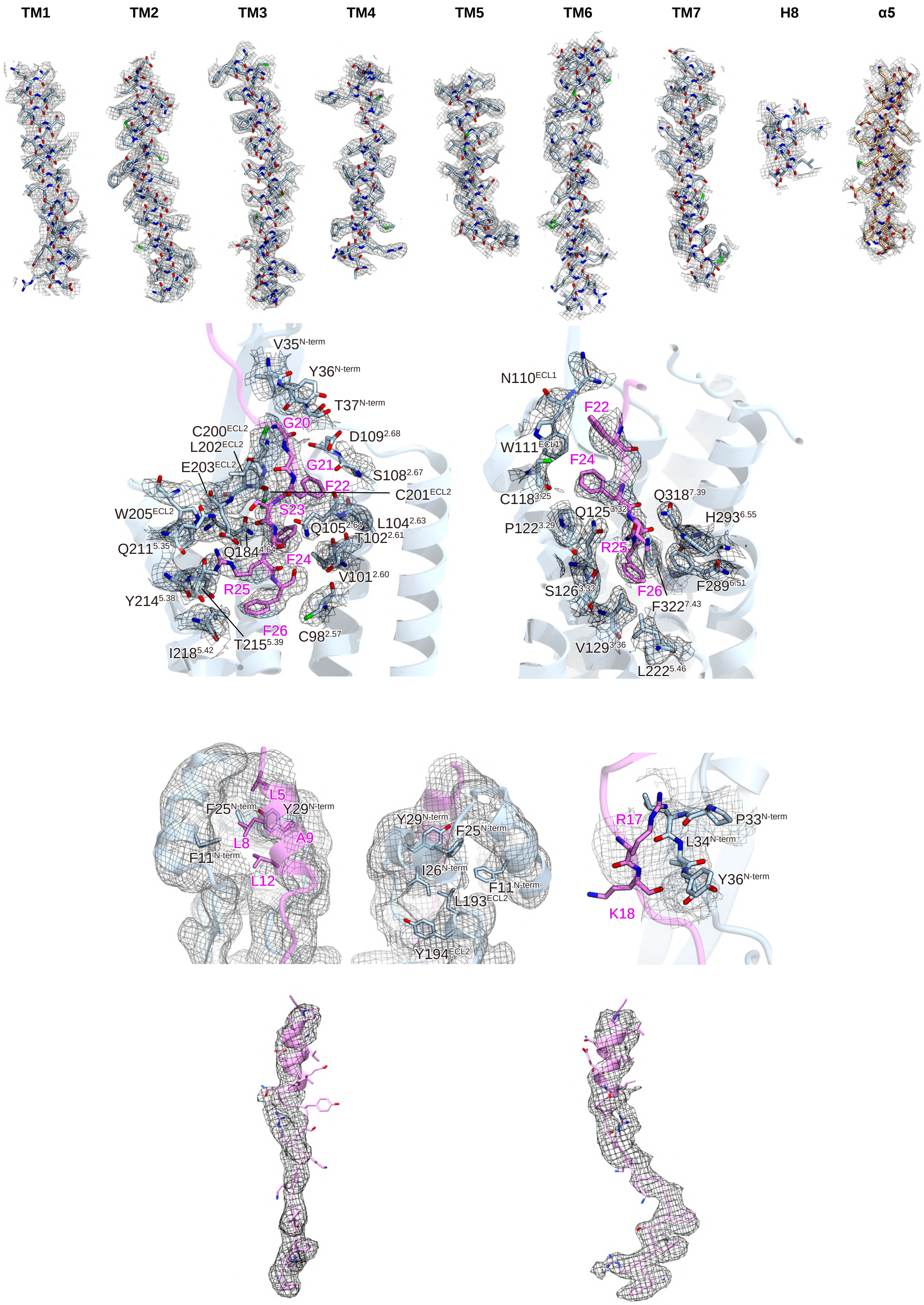

Supplementary Figure 3 | Cryo-EM analysis.
Flow chart of the cryo-EM data processing for the GPR103-miniG_q complex, including particle projection selection, classification, and 3D density map reconstruction. The 3D density map was refined with a mask on the receptor. Details are provided in the Methods section. The densities corresponding to each receptor part in the enhanced map are shown.

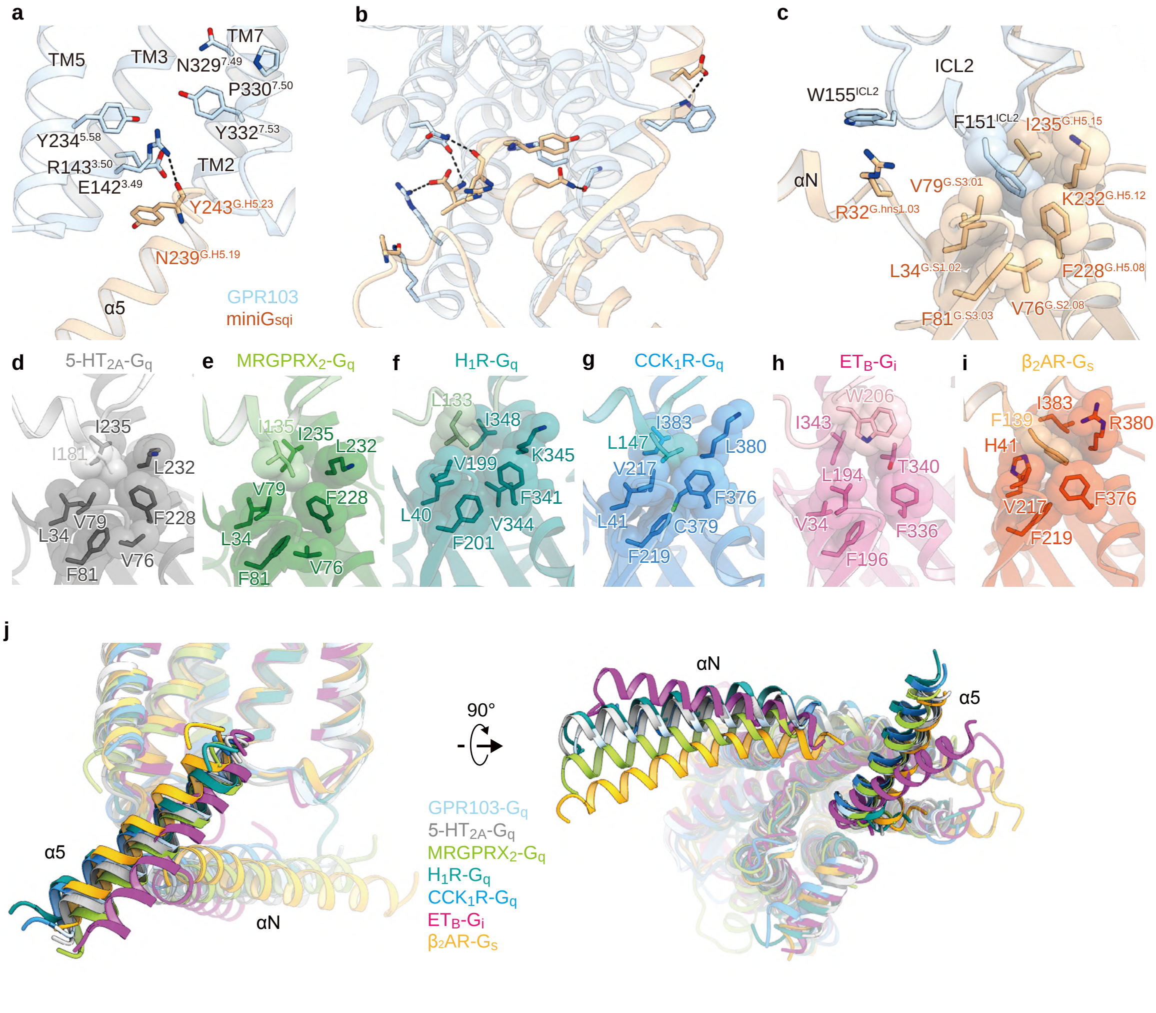

Supplementary Figure 4 | The Gq Binding mode.

a, b Interface between GPR103 (blue) and α5h of Gα_q (orange) shown with critical residues and polar interactions represented by stick models and dotted lines. The C-terminal helix of Gα_q (α5h) is deeply embedded within the intracellular cavity. In the proximity of the widely conserved $N^{7.49}P^{7.50}xxY^{7.53}$ motif19, the side chains of Y234$^{5.58}$ and Y332$^{7.53}$ are oriented towards each other. Around another conserved $D^{3.49}R^{3.50}Y^{3.51}$ motif (modified to ERH in GPR103), R$^{143 3.50}$ forms a hydrogen bond with the backbone carbonyl of Y243$^{G.H5.23}$ (superscript indicates the common Gα numbering [CGN] system20) in the α5h of Gα_q. **c** Interface between ICL2 of GPR103 and Gq shown with residues involved in hydrophobic interactions represented by CPK models. The bulky hydrophobic residue F151^{ICL2} is captured within a hydrophobic pocket in the Gα subunit (Fig. 2c). The other is stabilization through stacking interaction: W155^{ICL2} forms π-π stacking and π-cation interactions with R32$^{G.hns1.03}$. **d–i** Interface between ICL2 and G$_\alpha$ in other GPCR-G-protein complexes such as 5-HT$_{2A}$-G$_q$ (PDB 6WHA, gray), MRGPRX$_2$-G$_q$ (PDB 7S8N, yellow green), H$_1$R-G$_q$ (PDB 7DFL, dark cyan), CCK$_1$R-G$_q$ (PDB 7MBY, dodger blue), ETB-G$_i$ (PDB 8IY5, magenta), and β$_2$AR-G$_s$ (PDB 3SN6, yellow orange). This comparison elucidates that the hydrophobic interaction between ICL2 and G-protein is a common structural feature of the complex assembly. **j** Comparison of the angles and positions of α5h and αN relative to the receptor. In terms of the binding angles for α5h, GPR103-G$_q$ naturally exhibits similarity to G$_q$-coupled GPCRs rather than G$_s$- and G$_i$-coupled GPCRs

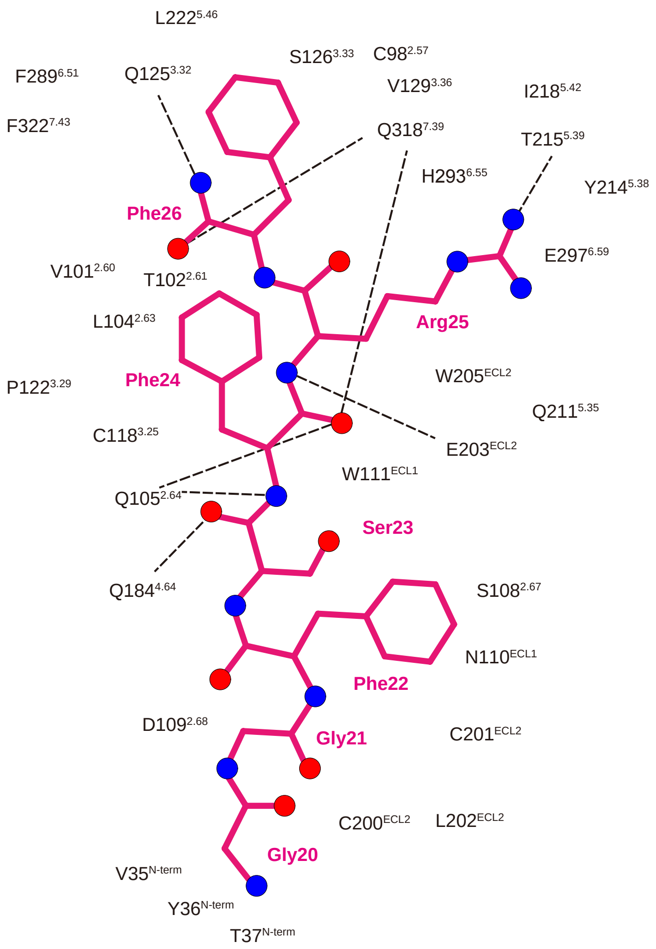

Supplementary Figure 5 | Binding site diagram.
Residues within 4.5 Å of the ligand are analysed by LIGPROT. Black dashed lines indicate hydrogen bonds.

a

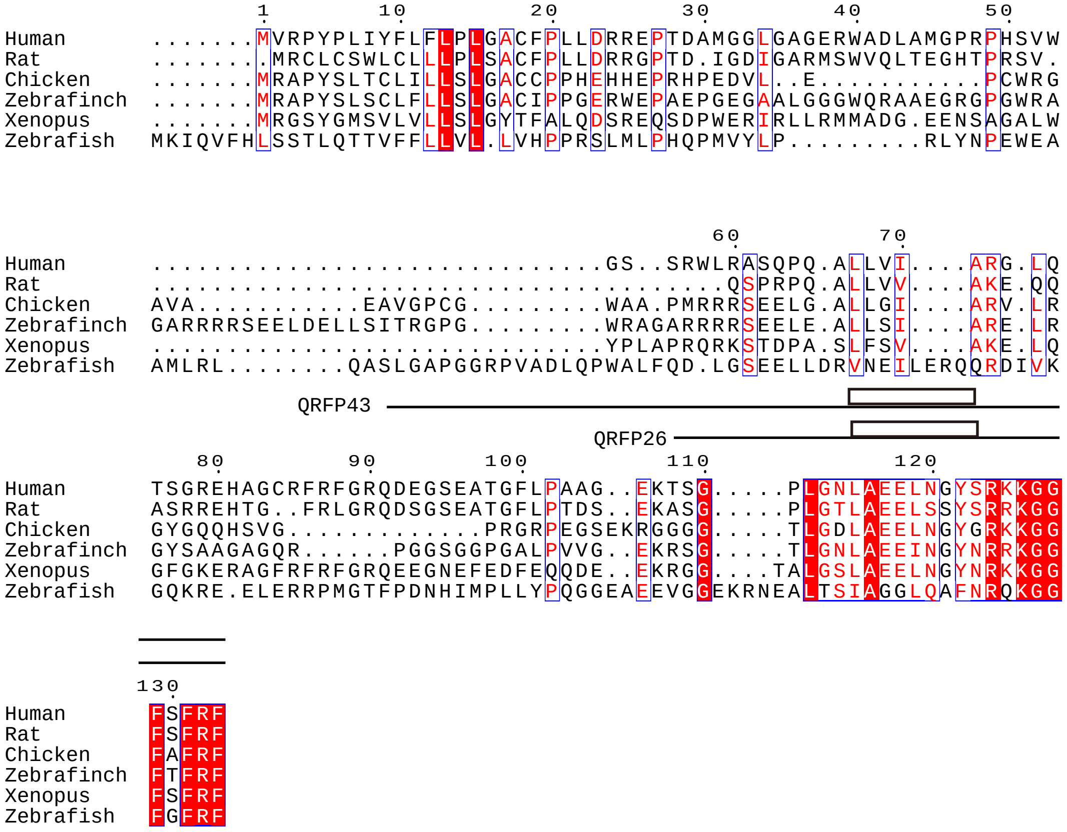

b

No.	35	36	37	98	101	102	104	105	108	109	110	111	118	122	125	126	129
BW	N-term	N-term	N-term	2.57	2.6	2.61	2.63	2.64	2.67	2.68	23.49	23.5	3.25	3.29	3.32	3.33	3.36
Human	V	Y	T	C	V	T	L	Q	S	D	N	W	C	P	Q	S	V
Rat	V	Y	T	C	V	T	L	Q	S	D	K	W	C	P	Q	S	V
Chicken	V	Y	V	C	F	T	L	Q	S	S	E	W	C	P	Q	S	I
Zebrafinch	V	Y	I	C	F	T	L	Q	S	S	N	W	C	P	Q	S	I
Xanopus	V	Y	I	C	F	T	L	Q	S	S	N	W	C	P	Q	S	I
Zebrafish	V	Y	I	C	F	T	L	Q	S	S	E	W	C	P	Q	T	V

No.	184	200	201	202	203	205	211	214	215	218	222	289	293	297	318	322
BW	4.64	ECL2	ECL2	ECL2	ECL2	ECL2	5.35	5.38	5.39	5.42	5.46	6.51	6.55	6.59	7.39	7.43
Human	Q	C	C	L	E	W	Q	Y	T	I	L	F	H	E	Q	F
Rat	Q	C	C	L	E	W	Q	Y	S	I	L	F	H	E	Q	F
Chicken	Q	C	C	L	E	W	Q	Y	T	I	L	F	H	E	Q	F
Zebrafinch	Q	C	C	L	E	W	Q	Y	T	I	L	F	H	E	Q	F
Xanopus	Q	C	C	L	E	W	Q	Y	T	I	L	F	H	E	Q	F
Zebrafish	Q	C	C	Q	E	W	R	Y	A	I	L	F	H	E	Q	F

c

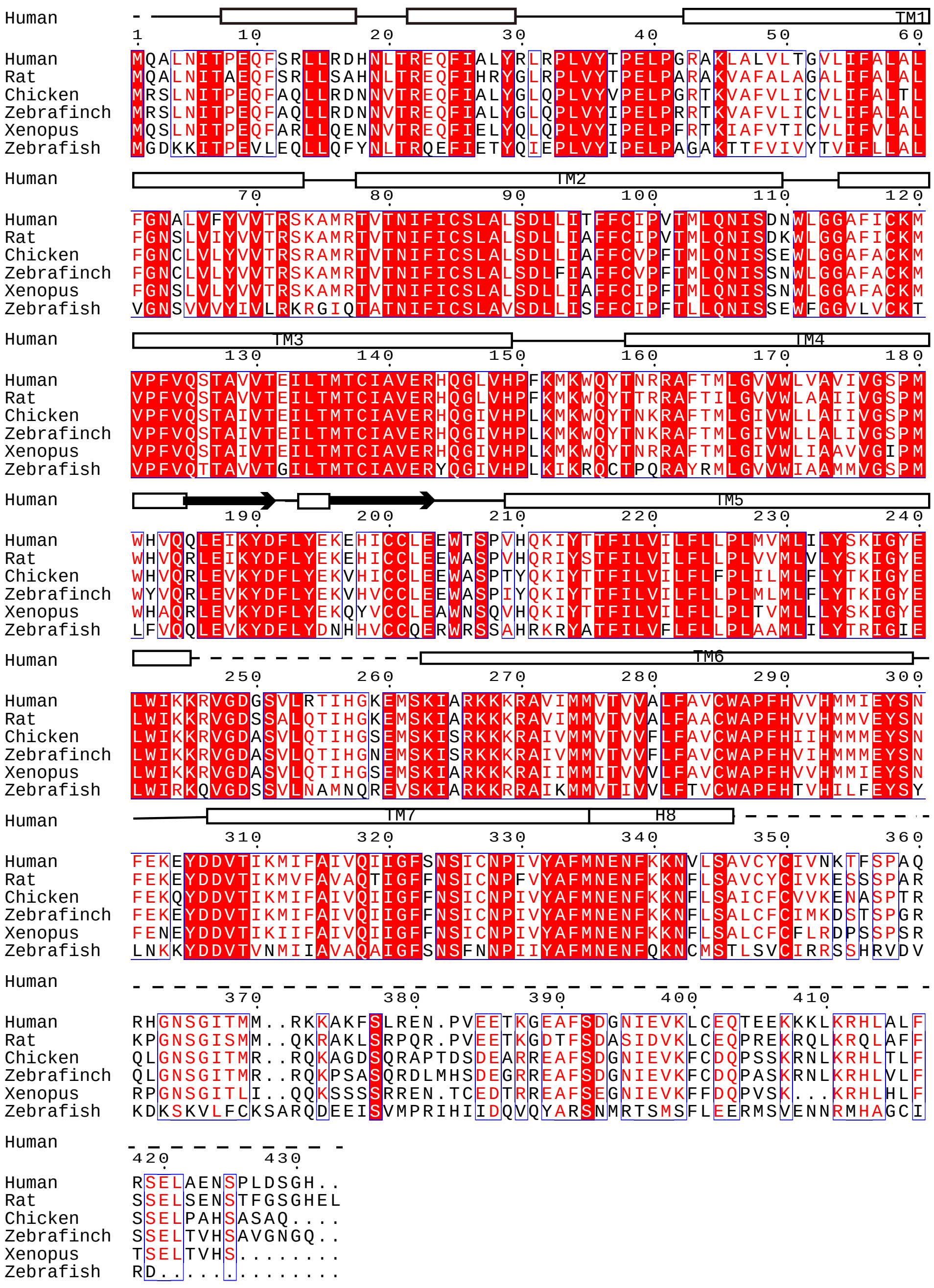

Supplementary Figure 6 | Conservation of the interaction site.
a Alignment of the amino acid sequences of QRFP homologs. **b** Comparison of residues within the TMD involved in QRFP binding in GPR103 homologues. **c** **A**lignment of the amino acid sequences of GPR103 homologs.

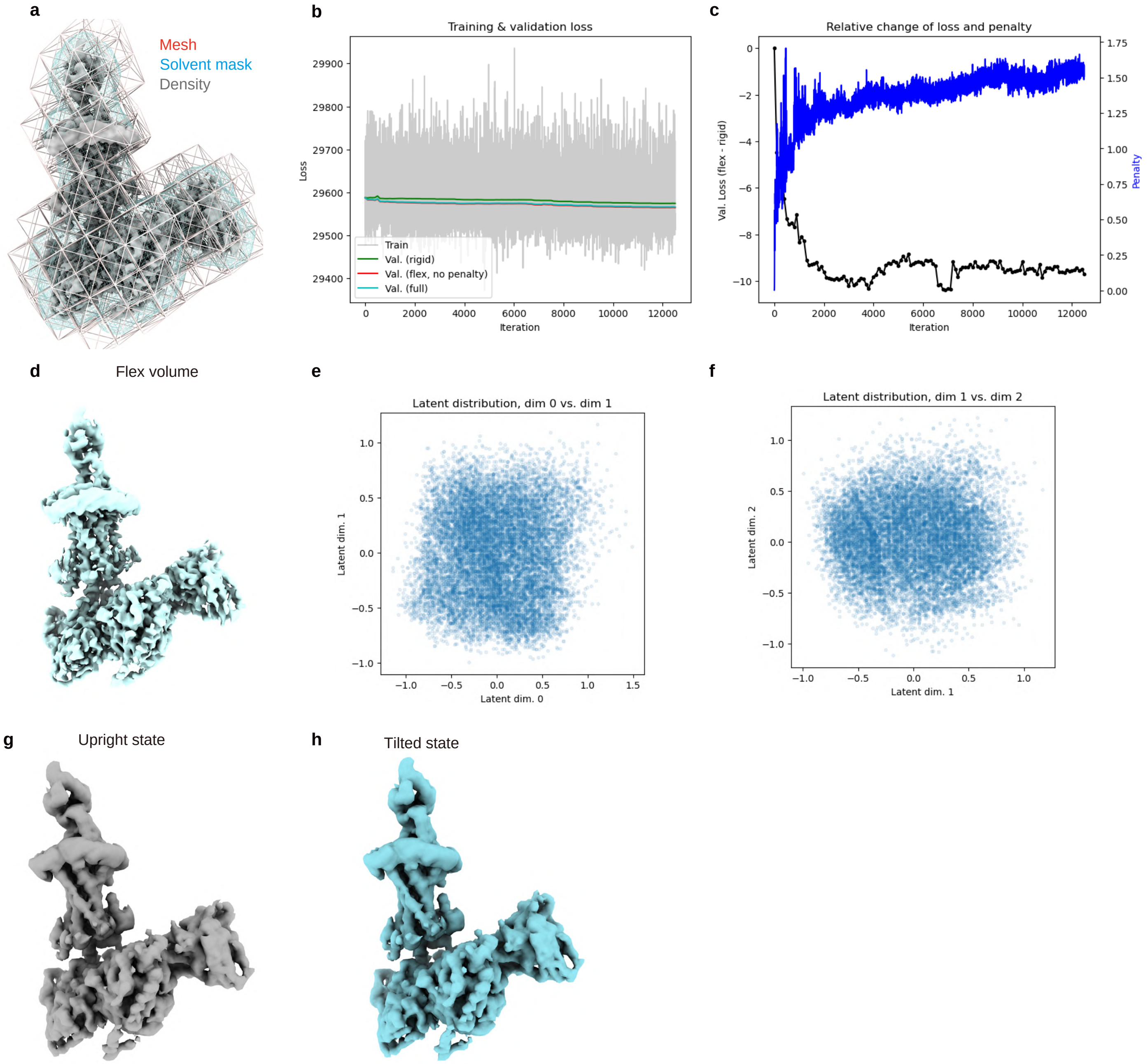

Supplementary Figure 7 | 3DFlex refinement model.

a A tetrahedral mesh, solvent mask and corresponding density which are used for 3DFlex. **b** Value of loss for each iteration. **c** In model validation, improvements in 3DFlex refinement compared to the non-3DFlex map through training iterations are shown in black, and changes in measured penalty compared to the unmodified map are shown in blue. **d** Flex volume: an output of high resolution reconstruction for 3DFlex. **e, f** Latent distribution of each dimensions describing the conformational heterogeneity in the particle set. **g, h** Representative of the volume series output of the 3DFlex generator job. Of the 40 classes corresponding to the largest eigenvalues, frame0 and frame40 were defined as the upright and tilted states, respectively.d cpk models.

a

```
QRFP           EDEGSEATGFLPAAGEKTSGPLGNLAEELNGYSRKKGGFSFRF
RFRP-1                                     MPHSFANLPLRF
Kisspeptin-10                               YNWNSFGLRF
NPFF                                            FLFQPQRF
PrRP-31                    SRTHRHSMEIRTPDINPAWYASRGIRPVGRF
NPY                  YPSKPDNPGEDAPAEDMARYYSALRHYINLITRQRY
CCK                       KAPSGRMSIVKNLQNLDPSHRISDRDYMGWMDF
orexinB                      RSGPPGLQGRLQRLLQASGNHAAGILTM
```

b

```
GPR103

GPR103  ............................................................
GPR10   ............................................................
GPR54   ............................................................
GPR74   MNSFFGTPAASWCLLESDVSSAPDKEAGRERRALSVQQRGGPAWSGSLEWSRQSAGDRRR
GPR147  ............................................................

GPR103
                                                  1          10
GPR103  ..........................................M...QALNIT.PEQFSRL
GPR10   ............................MASSTTRGPRVSDLFSGLPPAVTTPANQSAEA
GPR54   ............................MHTVATSGPNA.........SWGAPANASGCP
GPR74   LGLSRQTAKSSWSRSRDRTCCCRRAWWILVPAADRARRERFIM...NEKWDTNSSENWHP
GPR147  ..........................................M...EGEPSQPPNSSWPL

GPR103                                        TM1
            20        30        40        50        60        70
GPR103  LRDHNLTREQFIALYRLRPLVYTPELPGRAKLALVLTGVLIFALALFGNALVFYVVTRSK
GPR10   SAGNGSVAGADAP..AVTPFQS.LQLVHQLKGLIVLLYSVVVVVVGLVGNCLLVLVIARVR
GPR54   GCGAN...ASDGP..VPSPR.....AV..DAWLVPLFFAALMLLGLVGNSLVIYVICRHK
GPR74   IWNVNDTKHHLYSDINITYVNYYLHQPQ.VAAIFIISYFLIFFLCMMGNTVVCFIVMRNK
GPR147  ..SQNGTNTEATPATNLTFSSYYQHTSP.VAAMFIVAYALIFLLCMVGNTLVCFIVLKNR

GPR103               TM2                             TM3
            80        90       100       110       120       130
GPR103  AMRTVTNIFICSLALSDLLITFFCIPVTM.LQNISDNWLGGAFICKMVPFVQSTAVVTEI
GPR10   RLHNVTNFLIGNLALSDVLMCTACVPLTLAYAFEPRGWVFGGGLCHLVFFLQPVTVYVSV
GPR54   PMRTVTNFYIANLAATDVTFLLCCVPFTA.LLYPLPGWVLGDFMCKFVNYIQQVSVQATC
GPR74   HMHTVTNLFILNLAISDLLVGIFCMPITL.LDNIIAGWPFGNTMCKISGLVQGISVAASV
GPR147  HMHTVTNMFILNLAVSDLLVGIFCMPTTL.VDNLITGWPFDNATCKMSGLVQGMSVSASV

GPR103                               TM4
           140       150       160       170       180       190
GPR103  LTMTCIAVERHQGLVHPFKMKWQYTNRRAFTMLGVVWLVAVIVGSPMWHVQQ..LEIKYD
GPR10   FTLTTIAVDRYVVLVHPLRRR..ISLRLSAYAVLAIWALSAVLALPAAVHTY.......H
GPR54   ATLTAMSVDRWYVTVFPLRALHRRTPRLALAVSLSIWVGSAAVSAPVLA..........L
GPR74   FTLVAIAVDRFQCVVYPFKPKL..TIKTAFVIIMIIWVLAITIMSPSAVMLHVQEEKYYR
GPR147  FTLVAIAVERFRCIVHPFREKL..TLRKALVTIAVIWALALLIMCPSAVTLTVTREEH.H

GPR103                                        TM5
               200       210       220       230       240
GPR103  FLYE.....KEHICCLEEWTSPVHQKIYT.TFILVILFLLPLMVMLILYSKIGYELWIKK
GPR10   ....VELKPHDVRLCEEFWGSQERQRQLYAWGLLLVTYLLPLLVILLSYVRVSVKLRNRV
GPR54   ....HRLSPGPRAYCSEAFPSRALERAFALYN.LLALYLLPLLATCACYAAMLRHLGRVA
GPR74   VRLNSQNKTSPVYWCREDWPNQEMRKIYT.TVLFANIYLAPLSLIVIMYGRIGISLFRAA
GPR147  FMVDARNRSYPLYSCWEAWPEKGMRRVYT.TVLFSHIYLAPLALIVVMYARIARKLCQAP

GPR103                                    TM6
          250       260       270       280       290       300
GPR103  RVGDGS..VLRTIHGKEMSKIARKKKRAVIMMVTVVALFAVCWAPFHVVHMMIEYSNFEK
GPR10   VPGC......VTQSQADWDRAR..RRRTFCLLVVIVVVFAVCWLPLHVFNLLRDLDPHAI
GPR54   VRPAPADSALQGQVLAERAGAV..RAKVSRLVAAVVLLFAACWGPIQLFLVLQALGPAGS
GPR74   VPHTGR......KNQEQWHVVSRKKQKIIKMLLIVALLFILSWLPLWTLMMLSDYADLSP
GPR147  GPAPGG......EEAAD.PRASRRRARVVHMLVMVALFFTLSWLPLWALLLLIDYGQLSA

GPR103                TM7                       H8
             310       320       330       340       350       360
GPR103  EYDD.VTIKMIFAIVQIIGFSNSICNPIVYAFMNENFKKNVLSAVCYCIVNKTFSPAQRH
GPR10   ...DPYAFGLVQLLCHWLAMSSACYNPFIYAWLHDSFREELRKL.LVAW.PRKIAPH...
GPR54   WHPRSYAAYALKTWAHCMSYSNSALNPLLYAFLGSHFRQAFRRV.CPCA.PRRPRRPRRP
GPR74   NELQ.IINIYIYPFAHWLAFGNSSVNPIIYGFFNENFRRGFQEAFQLQLCQKRAKPME.A
GPR147  PQLH.LVTVYAFPFAHWLAFFNSSANPIIYGYFNENFRRGFQAAFRARLCPRPSGSHKEA

GPR103
              370       380          390       400       410
GPR103  G..NSGITMMRKKAKFSLRE..NPVEETKGEAFSDGNIEVKLCEQTEEKKKLKRHLALFR
GPR10   ...GQN..MTV...SVVI..........................................
GPR54   GPSDPA..APH...AELLRLGSHPAPARAQKPGSSGLAARGLCVLGEDNA.........
GPR74   YALKAKSHVLINTSNQLVQE........ST..FQNPHGETLLYRKSAEKP..QQE..LVM
GPR147  YSERPG.GLLHRRVFVVVRP........SDSGLPSESGPSSGAPRPGRLP..LRNGRVAH

GPR103
       420       430
GPR103  SELAENSPLDSGH.........
GPR10   ......................
GPR54   .......PL.............
GPR74   EEL.KETTNSSEI.........
GPR147  HGLPREGPGCSHLPLTIPAWDI
```

Supplementary Figure 8| Sequence comparison of GPR103-related peptide receptors.
a Alignment of the amino acid sequences of GPR103-related peptides. **b** Alignment of the amino acid sequences of RF amide receptors.

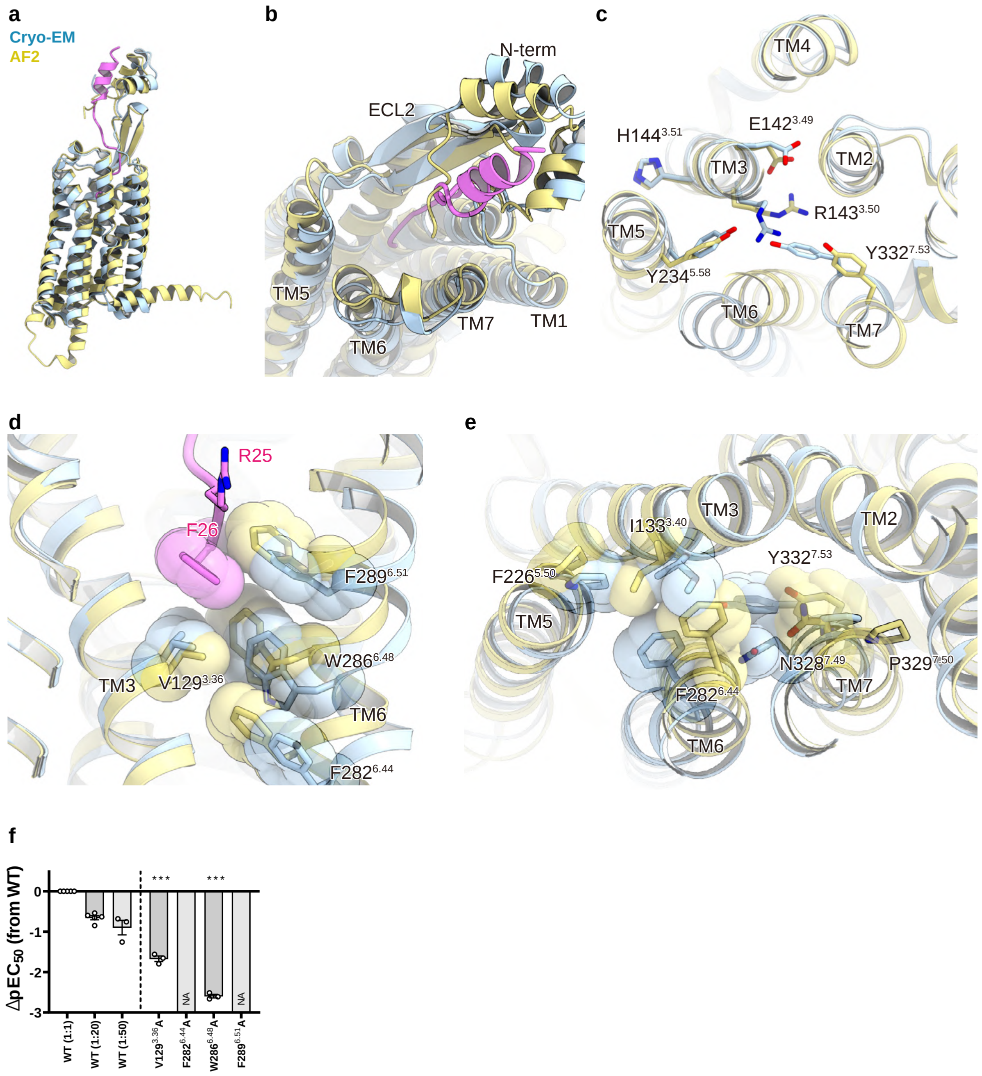

Supplementary Figure 9 | Comparison with AF2 model.

a–e Superimposition of the cryo-EM (blue) and AF2 structures (khaki), **(a)** overall view of the receptor, **(b)** focused on the extracelluar side, and **(c)** focused on the intracelluar side. Critical activation motifs are shown by the stick model. **(d)** Peptide-W648 interactions, and **(e)** PIF and NPxxY motifs. The important residues are shown in the stick and cpk models. **f** Effects of mutations in the ligand binding pocket of GPR103. QRFP26-induced activation of GPR103 was analyzed by the TGFα shedding assay. From the concentration–response curves (Supplementary Fig. 1a), ΔpEC50 values relative to the wild-type were calculated. Colors in the mutant bars indicate an expression level matching to that of titrated wild-type. NA, parameter not available because of lack of the ligand response. Statistical analyses were performed using the ordinary one-way ANOVA followed by Dunnett tests with the expression-matched (colored) WT response. ns, $p > 0.05$; *$p < 0.05$; **$p < 0.01$; ***$p < 0.001$. Data are presented as mean values ± SEM (*n*=3-5; dots).Source data are provided as a Source Data file.
