# Supplementary material for: Structure and dynamics of the pyroglutamylated RF-amide peptide QRFP receptor GPR103
Source: Nat Commun. 2024 Jun 19;15:4769. doi: 10.1038/s41467-024-49030-5 (PMC11187126; doi:10.1038/s41467-024-49030-5)
Supplement: Supplementary file 3 — Description of Additional Supplementary Files [file 41467_2024_49030_MOESM3_ESM.pdf]

File name: Supplementary Movie 1

Description: Conformational changes of GPR103 extracellular domain (ECD).
